# Risk factors associated with Para-Aortic Lymph Node Failure after pelvic irradiation in patients with Cervical Cancer

**DOI:** 10.7150/jca.45520

**Published:** 2020-06-28

**Authors:** Weiping Wang, Dunhuang Wang, Xiaoliang Liu, Yuncan Zhou, Jiabin Ma, Xiaorong Hou, Ke Hu, Fuquan Zhang

**Affiliations:** Department of Radiation Oncology, Peking Union Medical College Hospital, Chinese Academy of Medical Sciences & Peking Union Medical College, Beijing, China.

**Keywords:** cervical cancer, para-aortic lymph node failure, extended-field irradiation, indication

## Abstract

**Objective:** Previous studies have shown that prophylactic extended-field irradiation can reduce para-aortic lymph node failure (PALNF) rates in patients with cervical cancer. As such, this type of irradiation may particularly benefit patients with a high risk of PALNF. In the present study, we analyzed the risk factors for PALNF in patients with cervical cancer treated with pelvic irradiation in order to identify potential indications of prophylactic extended-field irradiation.

**Methods:** We evaluated patients with 2018 FIGO stage IB3-IIIC1 cervical cancer who were treated with definitive pelvic radiotherapy or concurrent chemoradiotherapy at our institution between 2011 and 2014. Univariate and multivariate analyses were performed to identify risk factors for PALNF.

**Results:** We included 572 patients in the study. The median follow-up period was 37.9 months. Eighteen patients (3.1%) first site of tumor relapse was the para-aortic lymph nodes, and thus showed PALNF. Using multivariate Cox regression analysis, we identified two significant risk factors for PALNF: tumor extension to the pelvic wall (hazard ratio, HR 3.60, p=0.026) and ≥ 2 pelvic MLNs (HR 5.30, p=0.005). For patients with and without risk factors, the 3-year overall survival, disease-free survival, and PALNF rates were 77.3% and 90.1% (p<0.001), 56.4% and 83.1% (p<0.001), and 12.0% and 2.3% (p<0.001), respectively.

**Conclusion:** Tumor extension to the pelvic wall and ≥ 2 pelvic MLNs are positively associated with PALNF after pelvic irradiation in patients with cervical cancer. Further trials will be required to validate whether patients with these two risk factors may benefit from prophylactic extended-field irradiation.

## Introduction

Cervical cancer is the most common gynecological carcinoma in women worldwide 1. In particular, locally advanced cervical cancer (LACC) accounts for a large proportion of patients in China, most likely because cervical cytologic screening methods have not been widely used in Chinese practice. The current standard treatment for patients with LACC is concurrent chemoradiotherapy (CCRT) globally. For LACC patients without para-aortic lymph node metastases, the standard radiation therapy treatment field is the pelvis. The para-aortic lymph node region is excluded from the treatment field, despite the fact that it plays an important role in cervical cancer metastasis and is one of the most common sites of tumor relapse 2, 3. To reduce the incidence of para-aortic lymph nodes failure (PALNF) and improve the survival of cervical cancer patients without para-aortic lymph node, para-aortic lymph nodes region is included in the target volume of radiation therapy in some institutes. This is prophylactic extended-field irradiation in patients with cervical cancer.

It is controversial whether prophylactic extended-field irradiation is beneficial for patients with LACC who were treated with CCRT 4-10. In our previous study, extended-field irradiation decreased para-aortic lymph node failure (PALNF) and distant failure rates in patients with cervical cancer. However, overall survival (OS) and disease-free survival (DFS) rates were not significantly different between prophylactic extended-field irradiation and pelvic radiotherapy, suggesting limited benefits of prophylactic extended-field irradiation 4. One reason for this finding is that, in this previous study of ours, a large proportion of patients were early-stage patients without high-risk factors 4, and probably only patients with advanced disease could benefit from prophylactic extended-field irradiation.

Considering prophylactic extended-field irradiation could reduce PALNF rates for patients with cervical cancer 4, 5, 10, it may be of particular benefit to patients with a high risk of PALNF. Currently, studies on the risk factors for PALNF after pelvic irradiation in patients with cervical cancer are limited. A Taiwanese study of 758 patients with cervical cancer treated with pelvic irradiation found that, after a median follow-up of 50 months, 80 patients (11%) showed PALNF. After the multivariate analysis, an SCC Ag level ≥ 40 ng/ml, advanced parametrial involvement, and pelvic metastatic lymph nodes (MLNs) were identified as independent factors positively associated with PALNF. The 5-year PALNF rates in patients with SCC Ag ≥ 40 ng/ml, parametrial score 4-6, pelvic MLNs, and no risk factor were 57%, 34%, 37%, and 9%, respectively^2^. In the present study, we analyzed the risk factors for PALNF in patients with cervical cancer treated with pelvic radiotherapy or CCRT in our institute to identify patients who may benefit from prophylactic extended-field irradiation.

## Materials and Methods

### Patients

We retrospectively reviewed the medical records of patients with cervical cancer treated with definitive radiotherapy or CCRT at our institute between January 2011 and December 2014. The inclusion criteria were as follows: biopsy-confirmed cervical cancer; 2018 International Federation of Gynecology and Obstetrics (FIGO) stage IB3 to IIIC1 11; and treatment with definitive pelvic radiation therapy or CCRT. Patients previously treated with extended-field irradiation were excluded. The stage of patients was recorded with 2009 FIGO staging system 12 and converted to 2018 FIGO stage 11.

### Treatment

The clinical target volume (CTV) for radiotherapy comprised the gross tumor, uterus, cervix, upper part of the vagina, parametrium and pelvic lymph node regions, with the latter comprising the common iliac, external iliac, internal iliac, obturator, and presacral lymph node regions. The upper border of the CTV was at the level of aortic bifurcation. The gross tumor volume (GTV) covered pelvic MLNs. The planning clinical target volume (PCTV) was defined as the CTV plus margins of 6-10 mm. Margins of 5 mm were added to the GTV to form the planning gross tumor volume (PGTV). For intensity-modulated radiation therapy (IMRT), a dose of 50.4 Gy in 28 fractions was prescribed to the PCTV, and the PGTV was simultaneously boosted to 59-61 Gy. Image guidance was used for the delivery of IMRT 3. Brachytherapy generally started after 3 weeks of IMRT. With high-dose-rate brachytherapy, 30-36 Gy in 5-7 fractions was delivered to point A. The first-line regimen of concurrent chemotherapy was 40 mg/m^2^ cisplatin weekly. The detailed treatment protocol was described previously 4, 13.

### Follow-up

Patients received a follow-up examination one month after the completion of treatment. Follow-up examinations were then conducted every three months during the first two years after treatment cessation, every six months during the next three years, and subsequently every year. These routine follow-up examinations included a gynecological examination, squamous cell carcinoma antigen (SCC Ag) measurement, pelvic MRI, and chest and abdominal CT. For patients with suspected tumor relapse, a tumor biopsy or positron emission tomography (PET)/CT was suggested.

### Statistics

A univariate analysis was performed with log-rank methods. A multivariate analysis was conducted with the Cox regression model. OS, DFS, and PALNF were calculated with Kaplan-Meier methods. Only the first site of relapse was evaluated. Tumor extension to the pelvic wall was diagnosed with gynecological examination, and the definition was the same with stage IIIB in 2009 FIGO staging system 11. All statistical analyses were performed with SPSS (version 23.0; SPSS, Inc., Chicago, IL, USA).

## Results

There were 723 patients with 2018 FIGO stage IB3-IIIC1 cervical cancer treated at our institute between January 2011 and December 2014. Of these patients, 151 were treated with extended-field irradiation, and were thus excluded. A total of 572 patients were therefore included in the present study. The basic demographic characteristics of the patients are shown in Table 1. There were 48 patients (8.4%) with tumor extension to the pelvic wall and 95 patients (16.6%) with FIGO stage IIIC1 disease. Common iliac lymph nodes metastasis was observed in 5 patients (0.9%).

**Figure 1:**
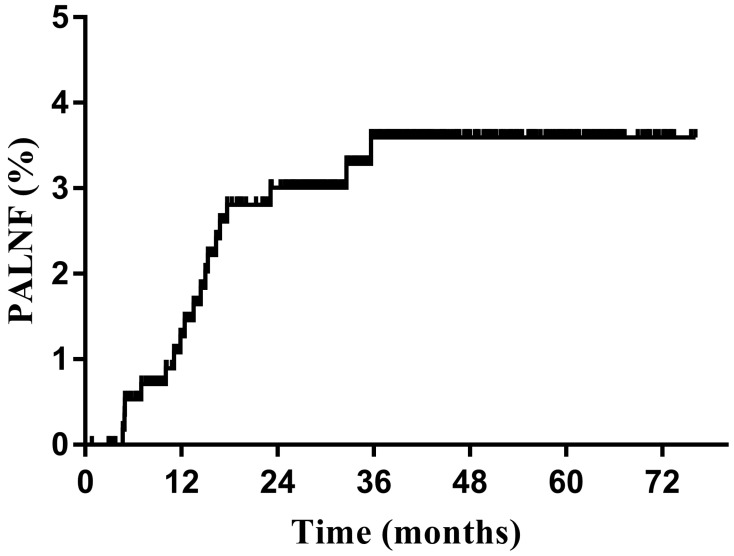
Para-aortic lymph node failure (PALNF) rates of patients with cervical cancer treated with pelvic irradiation (n=572).

The median follow-up period was 37.9 months (range, 1.0-76.2 months). The 3-year OS, DFS, and PALNF rates were 88.3%, 79.2%, and 3.6% (Figure 1), respectively. Eighteen patients (3.1%) experienced PALNF. The median time from the end of treatment to identification of PALNF was 14.0 months (range, 4.7-35.7 months). Of these 18 patients, 10 had only PALNF, while the other 8 had tumor relapse at other sites as well - 3 patients with pelvic lymph node relapse, 1 patient with vaginal recurrence, 1 patient with parametrial recurrence, 1 patient with lung metastasis, 1 patient with mediastinum metastasis and 1 patient with supraclavicular metastasis.

The univariate analysis of entire patient population revealed positive associations with PALNF for the 2018 FIGO stage of the tumor (p<0.001), tumor extension to the pelvic wall (p=0.015), the presence of common iliac MLNs (p=0.026) and the number of pelvic MLNs (p=0.001) (Table 2). Considering that tumor extension to the pelvic wall and the number of pelvic MLNs (0 vs. 1 vs. ≥2) were strongly correlated with 2018 FIGO stage (IB3-IIIA vs. IIIB vs. IIIC1), 2018 FIGO stage was not incorporated into the multivariate analysis. The multivariate analysis revealed two significant risk factors for PALNF: tumor extension to the pelvic wall (hazard ratio (HR) 3.60, 95% confidence interval, CI 1.17-11.10, p=0.026) and ≥ 2 pelvic MLNs (HR 5.30, 95% CI, 1.65-17.01, p=0.005) (Table 3).

**Figure 2:**
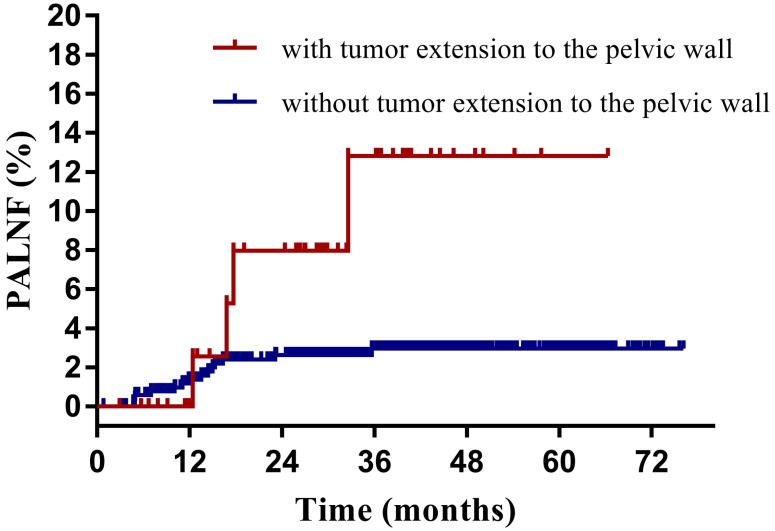
Para-aortic lymph node failure rates of cervical cancer patients with (n=48) and without tumor extension to the pelvic wall (n=524).

**Figure 3:**
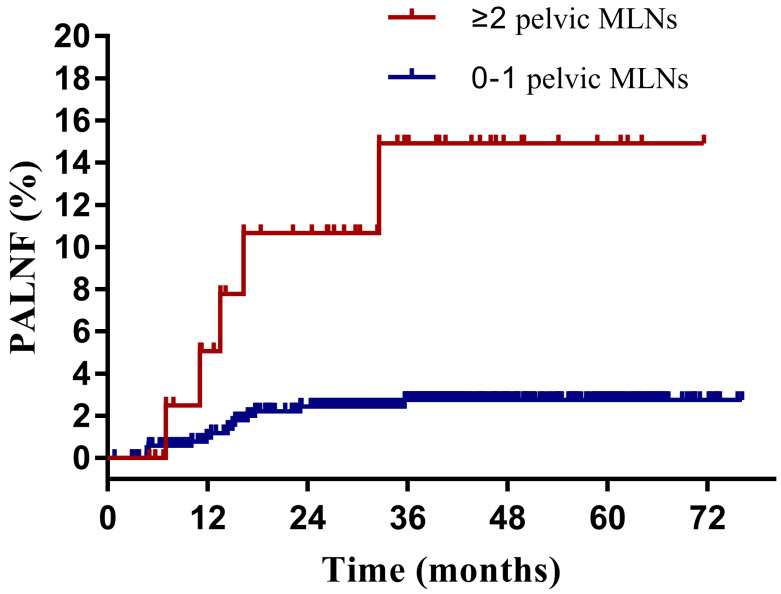
Para-aortic lymph node failure rates of cervical cancer patients with 0-1 (n=528) or ≥ 2 pelvic metastatic lymph nodes (MLNs, n=44).

**Figure 4:**
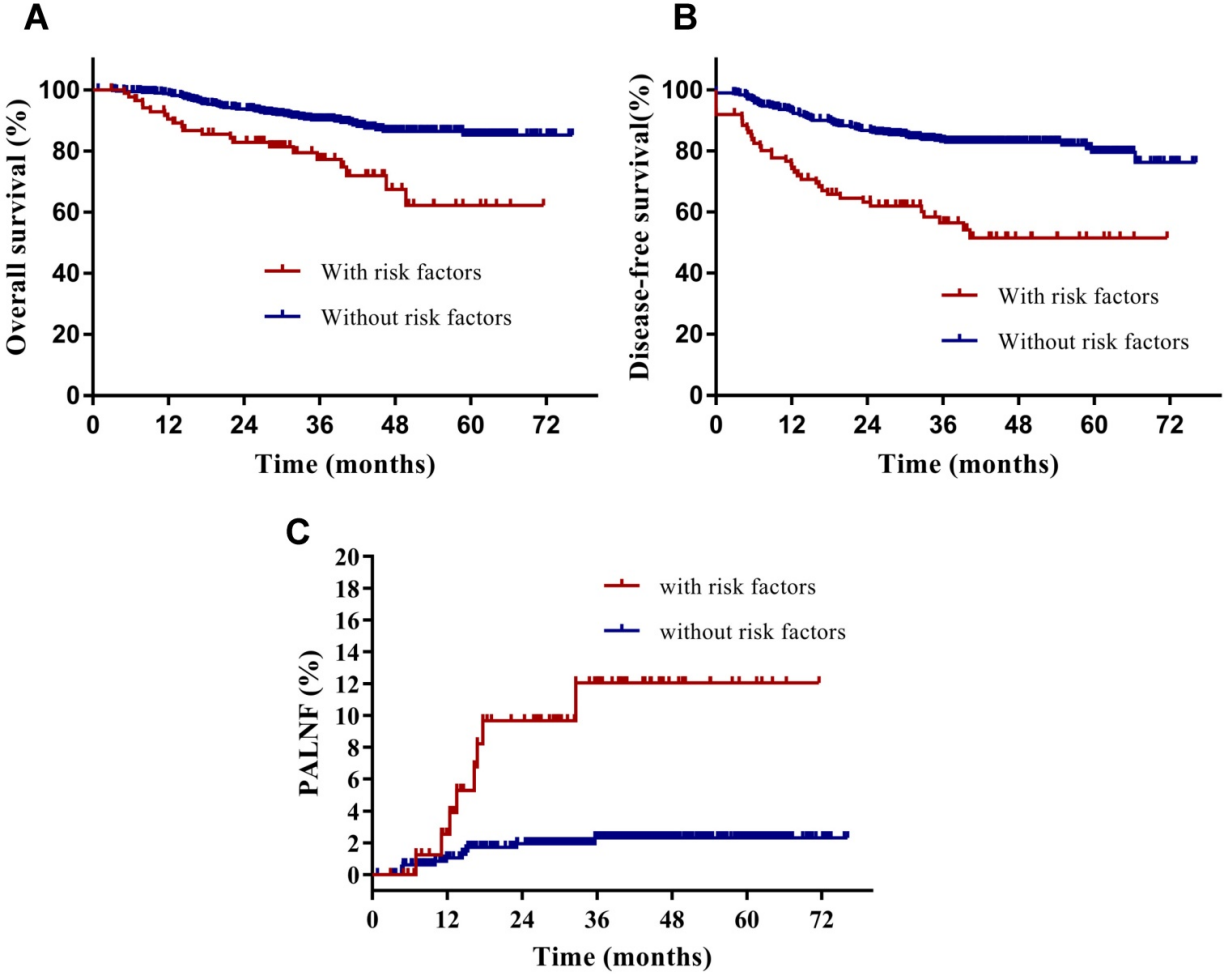
Disease-free survival (**A**), overall survival (**B**) and para-aortic lymph node failure (**C**) rates of cervical cancer patients with (n=86) and without identified risk factors (n=486) for PALNF.

Having identified them as two key risk factors for PALNF, we next examined OS, DFS, and PALNF according to tumor extension to the pelvic wall and the number of pelvic MLNs. Tumor extension to the pelvic wall and ≥ 2 pelvic MLNs were observed in 44 and 48 patients, respectively. For patients with and without tumor extension to the pelvic wall, the 3-year OS, DFS, and PALNF rates were 77.3% and 89.3% (p=0.003), 61.9% and 80.7% (p<0.001), and 12.8% and 3.0% (p=0.015, Figure 2), respectively. For patients with 0-1 pelvic MLNs versus ≥ 2 pelvic MLNs, the 3-year OS, DFS, and PALNF rates were 89.4% and 73.8% (p<0.001), 82.1% and 45.5% (p<0.001), and 2.8% and 14.9% (p<0.001, Figure 3), respectively. There were 486, 80, and 6 patients with none, one, or both of the defined risk factors, respectively. For patients with and without risk factors, the 3-year OS, DFS, and PALNF rates were 77.3% and 90.1% (p<0.001, Figure 4A), 56.4% and 83.1% (p<0.001, Figure 4B), and 12.0% and 2.3% (p<0.001, Figure 4C), respectively. For patients with one versus both risk factors, the 3-year OS, DFS, and PALNF rates were 79.3% and 50.0% (p=0.050), 60.1% and 16.7% (p=0.013), and 10.1% and 50.0% (p=0.286), respectively.

## Discussion

In the present study, tumor extension to the pelvic wall and ≥ 2 pelvic MLNs were associated with PALNF after pelvic irradiation in patients with LACC. Patients with these two factors may benefit from prophylactic EFI. These two identified risk factors for PALNF should be validated whether they are indeed predictive of the therapeutic success of prophylactic extended-field irradiation in patients with cervical cancer.

In our previous study, 133 patients with 2018 FIGO stage IIIB cervical cancer and treated with CCRT or radiotherapy were retrospectively analyzed. The 5-year OS and DFS of patients treated with pelvic radiotherapy and prophylactic extended-field irradiation were 66.3% and 80.3% (p=0.013), 57.2% and 80.4% (p=0.002), respectively, suggesting that patients with 2018 FIGO stage IIIB cervical cancer could benefit from cervical cancer^14^. It should be noted that tumor extension to the pelvic wall was not the same with stage 2018 FIGO stage IIIB. With 2018 FIGO staging system, patients with pelvic lymph nodes metastasis was classified to IIIC1. In the present study, part of the patients with tumor extension to the pelvic wall had positive pelvic lymph nodes. In the study of Lee et al, 206 patients with LACC and negative para-aortic lymph nodes who underwent pelvic radiotherapy (110 patients) and prophylactic extended-field irradiation (96 patients) were retrospectively reviewed. In subgroup analysis, compared with pelvic irradiation, prophylactic extended-field irradiation improved the OS of patients with FIGO III-IVA. In this study, most patients (32/47) with FIGO stage III-IVA cervical cancer had stage IIIB disease, indicating patients with FIGO stage IIIB cervical cancer could benefit from prophylactic extended-field irradiation 6.

It was reported that pelvic MLNs was associated with PALNF after pelvic radiotherapy in patients with cervical cancer 2, and prophylactic extended-field irradiation improved the OS of patients with pelvic MLNs 6. Studies focused on the number of pelvic MLNs and prophylactic extended-field irradiation was limited. In another study of Lee et al, 198 patients with LACC, pelvic MLNs and negative para-aortic lymph nodes were analyzed. Compared with pelvic radiotherapy, prophylactic extended-field irradiation significantly improved 5-year cancer-specific survival (56.5% vs. 96.3%, p<0.001) among patients with common iliac MLNs or ≥ 3 pelvic MLNs. For patients with pelvic MLNs below the common iliac bifurcation and 1-2 pelvic MLNs, the cancer-specific survival was not significantly different 15. In the present study, ≥ 2 pelvic MLNs was a risk factor of PALNF after pelvic irradiation. As we know, this factor has not been validated predictive of the therapeutic success of prophylactic extended-field irradiation.

We found that common iliac MLNs were not significantly associated with PALNF in patients with cervical cancer, likely because only 5 patients (0.9%) in our cohort presented with them. However, it has been previously reported that common iliac MLNs are associated with para-aortic MLNs 16, 17. Prophylactic extended-field irradiation improved the survival of patients with common iliac MLNs 15, and indeed the National Comprehensive Cancer Network (NCCN) guidelines also recommend that prophylactic extended-field irradiation be conducted for patients with common iliac MLNs. Therefore, common iliac MLNs are a clear indication of prophylactic extended-field irradiation, although our data could not draw that conclusion.

Tumor extensions to the pelvic wall and pelvic MLNs have been discussed extensively in patients with cervical cancer. It has been reported that these two factors are associated with poor survival in patients with cervical cancer 18-20. In the present study, only patients without para-aortic MLNs who were treated with pelvic radiotherapy were included. We found that tumor extension to the pelvic wall and ≥ 2 pelvic MLNs were associated with PALNF. This may potentially add some insight to the existing knowledge to this field.

The present study had some limitations. First, most patients with risk factors for PALNF had already received prophylactic extended-field irradiation and were therefore excluded from the study. As a result, compared with that of the primary population 4, the percentage of patients with risk factors for PALNF was small. The number of events was also limited in the present study, which may have influenced the study conclusions. With such a small sample size, especially the small sample size in some subgroups, it is difficult to draw a conclusive statistical statement. In addition, the conclusion of the present study should be interpreted with caution. Second, we did not validate whether the two identified risk factors for PALNF are indeed predictive of the therapeutic success of prophylactic extended-field irradiation in patients with cervical cancer in the present study. Further studies will be required to assess whether this hypothesized predictive potential is accurate in an independent cohort, such as publicly available databases. In 2019, our institute started a multicenter, randomized, phase 3 trial comparing pelvic radiotherapy and prophylactic EFI in selected patients with cervical cancer treated with concurrent chemoradiotherapy in China (NCT03955367). In this trial, tumor extension to the pelvic wall and ≥ 2 pelvic MLNs was used as inclusion criteria.

In conclusion, tumor invasion of the pelvic wall and ≥ 2 pelvic MLNs are related to PALNF after pelvic irradiation in patients with cervical cancer. If verified in subsequent clinical trials, these associations may inform the determination of which patients may benefit from prophylactic extended-field irradiation.

## Figures and Tables

**Table 1:** Baseline demographic, clinical and treatment characteristics of patients with 2018 FIGO stage IB3-IIIC1 cervical cancer treated with pelvic radiation therapy with or without concurrent chemotherapy

Characteristic	Number of patients	Percentage of patients
**Age (years)**		
Median	52 (range, 26-88)	
< 65	508	88.8%
≥ 65	64	11.2%
**Histology**		
Squamous cell carcinoma	506	88.5%
Adenocarcinoma	52	9.1%
Adenosquamous cell carcinoma	10	1.7%
Others	4	0.7%
**2018 FIGO stage**		
IB3	35	6.1%
IIA	50	8.7%
IIB	335	58.6%
IIIA	18	3.1%
IIIB	39	6.8%
IIIC1	95	16.6%
**Primary tumor size**		
< 4 cm	227	39.7%
≥ 4 cm	345	60.3%
Tumor extension to the pelvic wall*		
Yes	48	8.4%
No	524	91.6%
**Common iliac MLNs**		
Yes	5	0.9%
No	567	99.1%
**Bilateral pelvic MLNs**		
Yes	37	6.5%
No	535	93.5%
**Number of pelvic MLNs**		
0	477	83.4%
1	51	8.9%
2	36	6.3%
≥ 3	8	1.4%
**Large pelvic MLNs (≥ 1.5 cm)**		
Yes	541	94.6%
No	31	5.4%
**Concurrent chemotherapy**		
Yes	484	84.6%
No	88	15.4%

Abbreviations: MLNs = metastatic lymph nodes; * Tumor extension to the pelvic wall was diagnosed with gynecological examination.

**Table 2:** Results of the univariate analysis for patients with cervical cancer treated with pelvic irradiation

Characteristic	3-year PALNF rate	P value
**Age (years)**		
< 65	3.8%	0.501
≥ 65	1.6%	
**Histology**		
Squamous cell carcinoma	3.6%	0.922
Nonsquamous cell carcinoma	3.2%	
**2018 FIGO stage**		
IB3-IIIA	1.7%	<0.001
IIIB	9.5%	
IIIC1	11.3%	
**Primary tumor size**		
< 4 cm	1.8%	0.108
≥ 4 cm	4.8%	
**Tumor extension to the pelvic wall**		
Yes	12.8%	0.015
No	3.0%	
**Common iliac MLNs**		
Yes	3.4%	0.026
No	20.0%	
**Number of pelvic MLNs**		
0	2.2%	0.001
1	8.8%	
≥2	14.9%	
**Large pelvic MLNs (≥ 1.5 cm)**		
Yes	11.2%	0.245
No	3.3%	
**Concurrent chemotherapy**		
Yes	3.8%	0.674
No	2.4%	

Abbreviations: MLNs = metastatic lymph nodes.

**Table 3:** Results of the multivariate analysis for patients with cervical cancer treated with pelvic irradiation

	PALNF	
Variable	HR (95% CI)	P value
**Tumor extension to the pelvic wall**		
No	Reference	0.026
Yes	3.60 (1.17-11.10)	
**Common iliac MLNs**		
No	Reference	
Yes	2.30 (0.25-21.21)	0.461
**Number of pelvic MLNs**		
0	Reference	
1	2.86 (0.79-10.39)	0.111
≥ 2	5.30 (1.65-17.01)	0.005

Abbreviations: MLNs = metastatic lymph nodes.
